# Clinicopathological features of incidental parotid lesions

**DOI:** 10.1186/s13005-021-00262-6

**Published:** 2021-03-23

**Authors:** Hassan Al-Balas, Zeyad A. Metwalli, Sarah Eberson, David M. Sada

**Affiliations:** 1 Michael E. DeBakey VA Medical Center, 2002 Holcombe Blvd, 77030 Houston, Texas USA; 2 Jordan University of Science and Technology, Irbid, Jordan; 3 Baylor College of Medicine, One Baylor Plaza, 77030 Houston, Texas USA; 4 University of Texas M.D. Anderson Cancer Center, 1515 Holcombe Blvd, 77030 Houston, Texas USA

**Keywords:** Incidental parotid lesions, Asymptomatic parotid lesions, Parotid gland, Parotid neoplasm

## Abstract

**Background:**

The purpose of this study is to determine the histopathological spectrum and risk of primary malignancy of asymptomatic parotid lesions incidentally discovered on cross-sectional imaging.

**Methods:**

Over a 10-year period, 154 patients underwent 163 ultrasound-guided parotid lesion biopsies at our institution. This retrospective chart review included 89 lesions in 87 patients with asymptomatic parotid lesions discovered on cross-sectional imaging studies performed for unrelated clinical indications. The histopathologic findings of all sampled lesions were reviewed. We evaluated the patient demographics and pathological diagnoses of sampled parotid lesions to determine the histopathological spectrum and risk of malignancy.

**Results:**

The average age was 67.5 years and 92 % were males. 25 % of patients had bilateral lesions. The average size of the parotid lesions was 1.5 cm and 91 % were located in the superficial lobe. 92.1 % of lesions were benign with Warthin tumor being the most common diagnosis followed by pleomorphic adenoma. 2.3 % of lesions were primary parotid malignant neoplasms, while 5.6 % were metastatic lesions in patients with known malignancy.

**Conclusions:**

The incidence of primary parotid malignant neoplasm in asymptomatic incidentally discovered parotid lesions is low. Imaging or clinical follow-up may be considered in patients with incidental parotid lesions who prefer to avoid biopsy.

## Background

Parotid glands are the most common site of primary salivary gland neoplasms accounting for 70–85 % of these tumors. Although the majority of these tumors are benign, parotid glands still account for almost half of all malignant salivary gland tumors [1, 2]. Patients may present with a variety of symptoms including discomfort, pain, palpable mass, fullness and facial nerve palsy [1, 3–5]. With liberal use of cross-sectional imaging including computed tomography (CT), magnetic resonance imaging (MRI) and positron emission tomography (PET) to evaluate the head and neck, asymptomatic incidental parotid lesions are detected frequently. Although certain imaging characteristics may allow for non-invasive characterization of parotid lesions, tissue sampling is often needed for accurate diagnosis. Few previous studies have reported pathologic findings of incidental parotid lesions detected on PET imaging [6–8]. However, the clinical significance of asymptomatic parotid lesions detected incidentally by other more frequently utilized imaging modalities remains unclear.

The aim of this study is to retrospectively review the histopathologic findings of incidentally detected asymptomatic parotid lesions in a tertiary Veterans Affairs hospital to determine their clinical significance and risk of malignancy.

## Methods

Institutional ethical board review was obtained for this retrospective chart review study. Informed consent was not needed for this retrospective analysis.

Using an interventional radiology departmental procedure log, all patients who underwent image-guided parotid lesion biopsy at a tertiary Veterans Affairs hospital between January 2010 and December 2019 were identified. Based on the clinical indication for the biopsy, patients who were referred for biopsy because of symptoms related to the parotid gland lesions were excluded. Patients who underwent fine needle aspiration (FNA) or core biopsy of incidentally detected parotid lesions were reviewed. The hospital electronic medical record including the Computerized Patient Record System (CPRS) and Veterans Health Information Systems and Technology Architecture (VISTA) Imaging systems were used to review patient demographics and lesion features as well as histologic diagnoses. The patient demographics, lesion characteristics including size and location within the parotid gland, histopathologic findings and presence of previous head and neck malignancy were recorded. Statistical descriptive analysis of the data was performed using SPSS (IBM, Armonk, New York, USA).

## Results

Between January 2010 and December 2019, a total of 163 ultrasound-guided biopsies of parotid lesions were performed on 154 patients. Nine patients underwent bilateral parotid lesions biopsy. Of the lesions sampled, 89 asymptomatic incidentally detected parotid lesions were biopsied in 87 patients, which constituted the study population. The majority of lesions were sampled under ultrasound guidance utilizing 22-gauge fine needle aspiration. This technique was utilized exclusively in sampling 83 lesions. Additional core biopsy samples were obtained per pathologist request for three lesions. Three lesions were sampled using core biopsy needles without FNA. Core biopsy sampling was performed using 18-gauge core biopsy needles.”

The average age of study patients who underwent percutaneous biopsy of asymptomatic parotid lesions was 67.5 years (range 37–92 years). 80 patients (92 %) were males. 18.4 % of patients (16 patients) had a history of head and neck malignancy. Table 1 Summarizes patients’ demographics. 65 patients (75 %) had unilateral lesions and 22 patients (25 %) had bilateral lesions. Only two patients with bilateral parotid lesions underwent biopsy of bilateral lesions.

**Table 1:** Patient demographics

Characteristic	Total *N* = 87 (%)
Age, range (years)	67.5, 37–92
Gender	
Male	80 (92)
Female	7 (8)
History of head and neck malignancy	16 (18.4)
Laterality	
Unilateral	65 (75)
Bilateral	22 (25)

Most lesions were located in the superficial lobe of the parotid gland (81 lesions, 91 %) and the rest were located in the deep lobe (8 lesions, 9 %). The average size of the lesions was 1.5 cm (range 0.5–3.5 cm). 20 lesions were less than 1 cm (22.5 %), 59 lesions (66.3 %) were between 1 and 2 cm and 10 lesions (11.2 %) were larger than 2 cm. Table 2 summarizes the parotid lesion characteristics.

**Table 2:** Parotid lesion characteristics

Characteristic	Total *N* = 89 (%)
Tumor size (cm), range	1.5, 0.5–3.5
Location	
Superficial lobe	81 (91)
Deep lobe	8 (9)
Imaging modality which identified lesion	
CT (brain, sinus, neck)	47 (52.8)
MRI (brain, neck)	26 (29.2)
PET	16 (18)
Surgically resected	17 (19.1)

Table 3 summarizes the histopathologic findings. Warthin tumor was the most common pathology (45 lesions, 50.6 % of cases) followed by pleomorphic adenoma (15 patients, 16.9 %). Primary malignant parotid tumors accounted for only 2 lesions (2.3 % of the cases) and 5 lesions (5.6 %) represented metastatic disease (1 metastatic lesion from lung primary and 4 lesions were lymphomas with involvement of intra-parotid lymph nodes, with 3 of these patients having a prior history of lymphoma). All malignant lesions were greater than 1 cm in diameter.

**Table 3:** Histopathologic findings

Pathology	Total *N* = 89 (%)
Benign	82 (92.1)
Warthin tumor	45 (50.6)
Pleomorphic adenoma	15 (16.9)
Benign/reactive lymph node	11 (12.4)
Benign cyst	8 (9)
Inflammatory	1 (1.1)
Other	2 (2.3)
Malignant	7 (7.9)
Lymphoma	4 (4.5)
Mucoepidermoid carcinoma	2 (2.3)
Metastasis	1 (1.1)

17 lesions (19.1 %) were surgically resected, all of which were greater than 1 cm in size. Table 4 summarizes the histopathologic findings of surgically resected lesions. There were discrepancies between results of FNA and final surgical pathology in three lesions, ultimately confirmed to be Warthin tumors. FNA was suggestive of malignancy in one lesion in whom surgical pathology after resection was consistent with a Warthin tumor. FNA was suggestive of a pleomorphic adenoma in the second lesion, while surgical pathology after resection was consistent with a Warthin tumor. In a third lesion, FNA material was inadequate for diagnosis and surgical resection revealed a Warthin tumor.

**Table 4:** Histopathologic findings of surgically resected lesions

Pathology	Total *N* = 17 (%)
Benign	14 (82.4)
Pleomorphic adenoma	7 (41.2)
Warthin tumor	6 (35.3)
Benign lesion/ Foreign body reaction	1 (5.9)
Malignant	3 (7.9)
Mucoepidermoid carcinoma	2 (11.8)
Lymphoma	1 (5.9)

## Discussion

Cross-sectional imaging including CT, MRI and PET are increasingly utilized for evaluation of head and neck clinical problems, increasing the probability of discovering asymptomatic parotid lesions. The prevalence of these lesions is not known on CT and MRI, however their prevalence on PET imaging has been reported in the range of 0.3–0.45 % with reported incidence of malignancy up to 32 % of these lesions [7, 9, 10]. Knowledge of the risk of malignancy in these incidental lesions will aid the interpreting radiologist and referring physician in counseling patients prior to referral for percutaneous biopsy.

The average size of the lesions was 1.5 cm in this retrospective review. The small size explains why these lesions remained asymptomatic. More than 90 % of these lesions were located in the superficial lobe of the parotids and almost a quarter of them were bilateral, matching previously published imaging characteristics of parotid lesions [11–13].

Warthin tumor was the most common histopathology found in our study accounting for more than 50 % of the cases followed by pleomorphic adenoma. This is contrary to the reported frequency of symptomatic parotid tumors in literature with pleomorphic adenoma as the most frequent benign tumor in the parotid gland [3, 14]. Bothe et al. described similar dominance of Warthin tumor in an asymptomatic parotid lesions case series where 10 out of 12 lesions were reported to be Warthin tumors [15]. This could be related to slower growth of Warthin tumors, therefore, more likely to remain asymptomatic. The high incidence of Warthin tumors in our study population could also be reflective of the demographics of a predominantly male Veterans Affairs cohort with a high prevalence of smoking, both of which are factors associated with the development of Warthin tumors [2, 16].

In our study, we found the overall risk of parotid malignancy to be 7.9 %. Primary salivary gland tumors accounted for 2.3 % and an additional 5.6 % of these lesions were metastatic involvement of the parotid gland predominantly intra-parotid lymph node involvement with lymphoma. Stein et al. described in his study a 3.8 % risk of malignancy in subset group of patients with their lesions discovered incidentally on imaging studies. Therefore, in patients with no known primary, the risk of malignancy in these incidental parotid lesions is low [17]. This low risk persists despite our study population amongst the highest risk group for primary parotid malignancy as being predominantly males and elderly with an average age of 67.5 years. This incidence of malignancy is similar to previously reported incidence of 3.1-6 % in asymptomatic patients undergoing parotidectomy for treatment of parotid lesions [17, 18].

Several previously published studies have demonstrated the high accuracy of fine needle aspiration technique in evaluating parotid lesions. The reported sensitivity for parotid lesion FNA ranges from 70 to 90 %, while reported specificity and overall accuracy are as high as 97 % and 95 %, respectively [19–21]. Core needle biopsy of parotid lesions has been shown to have greater sensitivity and specificity of 93 % and 100 %, respectively [19–21]. In our study, 3 lesions with discrepancy between the FNA and surgical pathology were identified. Although none of these lesions proved to be malignant, surgical resection may have been avoided if an accurate diagnosis had been obtained with percutaneous biopsy. It is unknown if sampling with core biopsy would have yielded the correct diagnosis; however, this could be considered if the pathology report after FNA biopsy is not conclusive.

## Conclusions

In our patient population, 2.3 % of asymptomatic, incidentally detected parotid lesions were found to represent a primary malignant parotid neoplasm. 5.6 % of asymptomatic parotid lesions sampled were reflective of lymphoma or metastatic disease. The low risk of malignancy identified in asymptomatic parotid lesions, particularly in the absence of known primary malignancy, may aid in the counseling of these patients regarding further management including the need for percutaneous biopsy or active surveillance with follow-up imaging. Although our study represents the largest collection of pathologically confirmed asymptomatic parotid lesions, additional larger scale studies with more diverse study population are needed to support follow up with imaging as a valid option in these patients and potentially avoid unnecessary biopsy.

## Data Availability

The collected data is stored in secure VA server and access to the data is restricted by federal regulation to the approved researchers and regulatory bodies because of personal identification information and privacy concerns. The data will be kept on the server according to the federal research policies.

## References

[1] Bjorndal K, Krogdahl A, Therkildsen MH (2011). Salivary gland carcinoma in Denmark 1990–2005: a national study of incidence, site and histology. Results of the Danish Head and Neck Cancer Group (DAHANCA). Oral Oncol.

[2] Pinkston JA, Cole P (1999). Incidence rates of salivary gland tumors: results from a population-based study. Otolaryngol Head Neck Surg.

[3] Spiro RH (1986). Salivary neoplasms: overview of a 35-year experience with 2,807 patients. Head Neck Surg.

[4] Venkatesh S, Srinivas T, Hariprasad S (2019). Parotid Gland Tumors: 2-Year Prospective Clinicopathological Study. Ann Maxillofac Surg.

[5] Quiriny M, Dekeyser C, Moreau M (2017). Benign tumors of the parotid gland: a retrospective study of 339 patients. Acta Chir Belg.

[6] Basu S, Houseni M, Alavi A (2008). Significance of incidental fluorodeoxyglucose uptake in the parotid glands and its impact on patient management. Nucl Med Commun.

[7] Lee SK, Rho BH, Won KS (2009). Parotid incidentaloma identified by combined 18F-fluorodeoxyglucose whole-body positron emission tomography and computed tomography: findings at grayscale and power Doppler ultrasonography and ultrasound-guided fine-needle aspiration biopsy or core-needle biopsy. Eur Radiol.

[8] Mabray MC, Behr SC, Naeger DM, Flavell RR, Glastonbury CM (2015). Predictors of pathologic outcome of focal FDG uptake in the parotid gland identified on whole-body FDG PET imaging. Clin Imaging.

[9] Makis W, Ciarallo A, Gotra A (2015). Clinical significance of parotid gland incidentalomas on (18)F-FDG PET/CT. Clin Imaging.

[10] Wang HC, Zuo CT, Hua FC (2010). Efficacy of conventional whole-body (1)(8)F-FDG PET/CT in the incidental findings of parotid masses. Ann Nucl Med.

[11] Joe VQ, Westesson PL (1994). Tumors of the parotid gland: MR imaging characteristics of various histologic types. AJR Am J Roentgenol.

[12] Stoia S, Baciut G, Lenghel M, et al. Cross-sectional imaging and cytologic investigations in the preoperative diagnosis of parotid gland tumors - an updated literature review. *Bosn J Basic Med Sci* 2020. 10.17305/bjbms.2020.5028 PMC7861630 32893758

[13] Mantsopoulos K, Tschaikowsky N, Goncalves M, Mueller SK, Iro H (2020). Evaluation of preoperative Ultrasonography in the Differentiation between Superficial and Deep Parotid Gland Tumors. Ultrasound Med Biol.

[14] Bradley PJ, McGurk M (2013). Incidence of salivary gland neoplasms in a defined UK population. Br J Oral Maxillofac Surg.

[15] Bothe C, Fernandez A, Garcia J (2015). Parotid incidentaloma identified by positron emission/computed tomography: when to consider diagnoses other than warthin tumor. Int Arch Otorhinolaryngol.

[16] Chung YF, Khoo ML, Heng MK, Hong GS, Soo KC (1999). Epidemiology of Warthin’s tumour of the parotid gland in an Asian population. Br J Surg.

[17] Stein AP, Britt CJ, Saha S (2015). Patient and tumor characteristics predictive of primary parotid gland malignancy: A 20-year experience at the University of Wisconsin. Am J Otolaryngol.

[18] Stodulski D, Mikaszewski B, Stankiewicz C (2012). Signs and symptoms of parotid gland carcinoma and their prognostic value. Int J Oral Maxillofac Surg.

[19] Gudmundsson JK, Ajan A, Abtahi J (2016). The accuracy of fine-needle aspiration cytology for diagnosis of parotid gland masses: a clinicopathological study of 114 patients. J Appl Oral Sci.

[20] Haldar S, Mandalia U, Skelton E (2015). Diagnostic investigation of parotid neoplasms: a 16-year experience of freehand fine needle aspiration cytology and ultrasound-guided core needle biopsy. Int J Oral Maxillofac Surg.

[21] Inohara H, Akahani S, Yamamoto Y (2008). The role of fine-needle aspiration cytology and magnetic resonance imaging in the management of parotid mass lesions. Acta Otolaryngol.

